# Progressive lung fibrosis: reprogramming a genetically vulnerable bronchoalveolar epithelium

**DOI:** 10.1172/JCI183836

**Published:** 2025-01-02

**Authors:** James P. Bridges, Eszter K. Vladar, Jonathan S. Kurche, Andrei Krivoi, Ian T. Stancil, Evgenia Dobrinskikh, Yan Hu, Sarah K. Sasse, Joyce S. Lee, Rachel Z. Blumhagen, Ivana V. Yang, Anthony N. Gerber, Anna L. Peljto, Christopher M. Evans, Elizabeth F. Redente, David W.H. Riches, David A. Schwartz

**Affiliations:** 1 Department of Medicine, National Jewish Health, Denver, Colorado, USA.; 2 Department of Medicine, Division of Pulmonary Sciences and Critical Care Medicine, University of Colorado Anschutz Medical Campus, Aurora, Colorado, USA.; 3 Rocky Mountain Regional Veteran Affairs Medical Center, Aurora, Colorado, USA.; 4 Department of Medicine, Division of Pulmonary and Critical Care Medicine, Stanford University, School of Medicine, Stanford, California, USA.; 5 Department of Pediatrics, University of Colorado Anschutz Medical Campus, Aurora, Colorado, USA.; 6 Department of Immunology and Genomic Medicine, National Jewish Health, Denver, Colorado, USA.; 7 Department of Biomedical Informatics, University of Colorado Anschutz Medical Campus, Aurora, Colorado, USA.; 8 Program in Cell Biology, Department of Pediatrics, National Jewish Health, Denver, Colorado, USA.; 9 Department of Immunology and Microbiology, University of Colorado Anschutz Medical Campus, Aurora, Colorado, USA.

## Abstract

Idiopathic pulmonary fibrosis (IPF) is etiologically complex, with well-documented genetic and nongenetic origins. In this Review, we speculate that the development of IPF requires two hits: the first establishes a vulnerable bronchoalveolar epithelium, and the second triggers mechanisms that reprogram distal epithelia to initiate and perpetuate a profibrotic phenotype. While vulnerability of the bronchoalveolar epithelia is most often driven by common or rare genetic variants, subsequent injury of the bronchoalveolar epithelia results in persistent changes in cell biology that disrupt tissue homeostasis and activate fibroblasts. The dynamic biology of IPF can best be contextualized etiologically and temporally, including stages of vulnerability, early disease, and persistent and progressive lung fibrosis. These dimensions of IPF highlight critical mechanisms that adversely disrupt epithelial function, activate fibroblasts, and lead to lung remodeling. Together with better recognition of early disease, this conceptual approach should lead to the development of novel therapeutics directed at the etiologic and temporal drivers of lung fibrosis that will ultimately transform the care of patients with IPF from palliative to curative.

Idiopathic pulmonary fibrosis (IPF) is a progressive lung disease, characterized by heterogeneous subpleural patches of fibrotic remodeled lung, that follows a bronchocentric distribution (1–3). The median survival is 3–5 years after diagnosis (1). While the etiology of IPF was initially unknown (thus, the nomenclature), we now understand that IPF is etiologically complex, with well-documented genetic and nongenetic origins. Lung fibrosis genetic risk variants demonstrate an autosomal dominant pattern of inheritance with incomplete penetrance (4), and in aggregate, these genetic risk variants account for at least 30% of the etiology of IPF (5). Cigarette smoke (6) and aging (7–9) also promote the development of IPF. How these nongenetic factors interact with specific genetic variants is not clear, but cigarette smoke and aging are known to contribute to epigenetic programming of the lung. Genetic susceptibility, epigenetic programming, and maladaptive homeostatic responses likely interact in ways that are yet to be described, reprogramming cells toward a fibroproliferative phenotype in the distal lung.

Genetic studies have identified dozens of rare and common genetic risk variants for IPF within key biological pathways that primarily affect the bronchiolar and alveolar epithelia (Table 1) (10). Although the gain-of-function *MUC5B* promoter variant is the dominant risk factor for this disease (11), accounting for at least 50% of the genetic risk of developing IPF (5), multiple biological mechanisms involving dysregulation of host defense, cell adhesion, telomere biology, mitotic spindle assembly, surfactant protein biology, and GTPase activity are implicated in the risk of developing IPF. Importantly, all genetic variants, except possibly a rare missense mutation in *SFTPC* (12), demonstrate incomplete penetrance for lung fibrosis, suggesting that ectopic expression or gain/loss of function of these genes establishes a biologically vulnerable phenotype that requires subsequent insults to trigger development of IPF.

Multiple types of environmental exposures promote the development of fibrotic interstitial lung disease (ILD; IPF is a type of ILD) and are candidate second hits within the appropriate genetic context. The dominant nongenetic factors that enhance the risk of IPF are aging (1, 7, 8, 13) and cigarette smoking (6, 14, 15), with each one-year increase in age associated with an approximately 6% increase in IPF prevalence (16) and cigarette smoking associated with an approximately 3- to 5-fold increase in the risk of IPF (4, 6). Aerosolized pollutants resulting from wildfires and other combustions, ozone, particulate matter (PM2.5 and PM10), metal dust, asbestos, farming, and livestock (14, 15, 17–19) have also been associated with interstitial lung abnormalities (considered a sign of early ILD or IPF, ref. 20), IPF incidence (21), and acute exacerbations of IPF (22–25). These nongenetic IPF risk factors suggest that mechanisms involving particle deposition (20, 25, 26), mucociliary dysfunction, epithelial injury with attendant persistent inflammation (27–29), stem cell exhaustion (30–32), and cell senescence (32–35) represent key drivers of the persistent fibrotic process. These risk factors may also be influenced by genetic variants. Such observations led to the two-hit hypothesis (36); in our model, the first hit establishes a vulnerable bronchoalveolar epithelium, and the second triggers mechanisms that reprogram distal epithelia to initiate and perpetuate a profibrotic phenotype (Figure 1).

In this Review, we discuss the two-hit hypothesis with an emphasis on MUC5B as the primary genetic risk factor, as it is an emerging aspect of IPF pathogenesis that has not been comprehensively addressed in prior reviews. We will also discuss how detrimental endoplasmic reticulum (ER) stress involving apoptosis, a persistent cycle of injury and repair, and activation of lung fibroblasts develop following additional damage to the terminal respiratory bronchiole. While IPF has been further characterized by dysregulation of immune cells and noncoding RNA signaling, these contributions are beyond the scope of the present discussion, and readers are directed elsewhere for comprehensive reviews of these topics (37, 38).

## Mechanisms initiating epithelial vulnerability

Peripheral remodeling and loss of alveolar gas exchange surfaces in IPF highlight a need to understand how vulnerable lung epithelial cells may be reprogrammed to perpetuate a profibrotic phenotype. Early work emphasized alveolar type II (ATII) cells as the main targets of injury and drivers of fibrosis. Recently, multipotent epithelial progenitors that give rise to both terminal airway and alveolar cells have been shown to be susceptible to injury and may contribute to fibrosis (39–51). When challenged with ongoing exposures, a rodent model demonstrated that epithelial progenitors fail to return to homeostasis and instead promote persistent injury and fibrosis through maladaptive repair, which was exacerbated in the context of enhanced MUC5B expression (29).

### Aberrant progenitors and regenerative epithelia.

**Figure 2:**
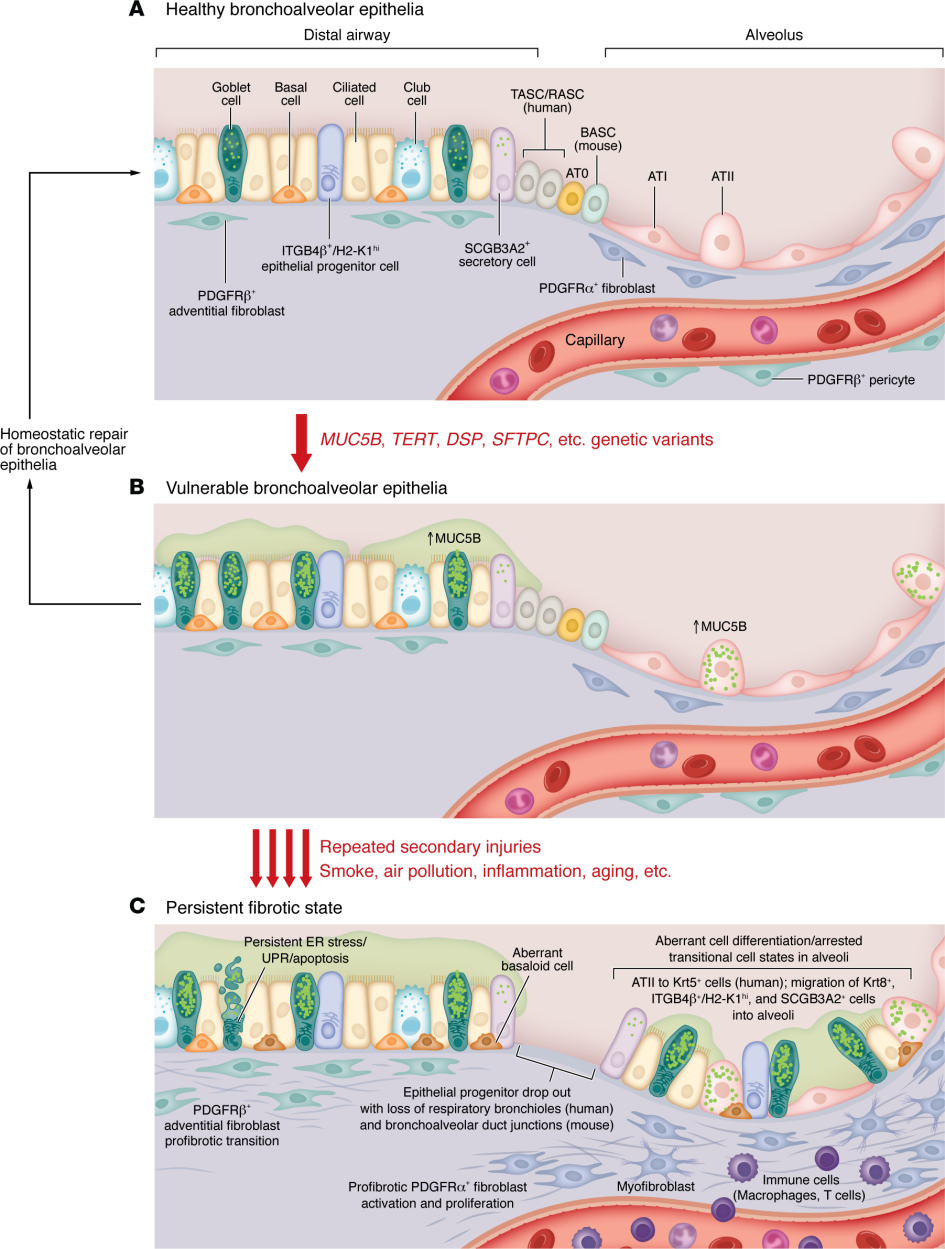
Model of the development of vulnerable bronchoalveolar epithelium as a contributing pathway to persistent pulmonary fibrosis. (**A**) In the healthy lung, the bronchoalveolar epithelium consists of proximal epithelial cells in the terminal airways (basal cells, ciliated cells, club cells, and goblet cells) and alveolar type II (ATII) and type I (ATI) cells in the alveoli and minimal if any expression of MUC5B. Identified epithelial progenitor populations, including ITGB4β^+^/H2-K1^hi^ cells in the conducting airways, BASCs at bronchoalveolar ducts in mice, and newly identified TASC, RASC, and AT0 cells in the preterminal and terminal respiratory bronchioles in humans, nonhuman primates, and ferrets are thought to be quiescent in the absence of injury. (**B**) In the presence of genetic variants (e.g., *MUC5B*), increased expression of MUC5B protein in goblet cells, and other cell types that do not typically express MUC5B protein (e.g., ATII cells), causes homeostatic ER stress, resulting in a vulnerable state that primes epithelial cell responses to subsequent injury. Repair of the bronchiolar and alveolar epithelia (**B**, left) is governed by epithelial cell/fibroblast/immune cell interactions near the site of injury that direct facultative epithelial progenitor cell (ATII) proliferation and differentiation into ATI cells and suppress fibroblast proliferation/activation. In addition, epithelial progenitor cells located at sites distant to the site of injury are activated and migrate to the injured alveolus (ITGB4β^+^/H2-K1^hi^ cells, BASCs) to restore formation of the air/blood barrier. However, in the context of repetitive secondary injuries (below **B**), the persistent and enhanced ER stress induces detrimental responses in the vulnerable epithelium, causing epithelial dysfunction during injury/repair, as indicated by aberrant epithelial cell differentiation, arrested transitional cell states, and activation of aberrant basaloid cells in the alveoli. (**C**) This leads to profibrotic fibroblast and pericyte activation, proliferation, and excess extracellular matrix deposition. The consequence of respiratory bronchiole dropout in patients with early-stage IPF and the role of RASCs, TASCs, and AT0 progenitor populations in homeostatic repair versus a persistent fibrotic state has yet to be determined.

Findings from murine and human studies demonstrate that fibrosis in IPF persists owing to sustained disruption of tissue homeostasis and recognize the central role of progenitor cells and cell populations in aberrant transitional states. The specific cell types and pathways involved in homeostatic repair and disease will likely depend on the model organism studied, owing to anatomical differences in the distal lungs among humans, nonhuman primates, and rodents (Figure 2). Yet, a subset of Wnt-responsive ATII cells proliferate in response to injury and differentiate into alveolar type I (ATI) cells to repair the alveolar epithelium following injury in mice (52, 53) and have also been shown to possess progenitor function in human organoid cultures. During fibrosis, ATII cells exhibit a transitional morphology and gene expression profile consistent with ineffectual/stalled differentiation to ATI cells (54, 55). In clustered, cystic airspaces termed honeycomb cysts in IPF, this transitional state is marked by expression of one or more keratin genes (KRT5 and KRT14) and in simple cysts, by KRT8 and KRT18 in the bleomycin mouse model (11, 56, 57). Genetic variants in the *KRT8* locus are associated with IPF, and KRT8^+^ epithelial cells have a direct pathologic role in driving fibroblast activation, proliferation, and collagen deposition in the bleomycin model (58). Molecular pathways associated with these transitional states currently include TGF-β, p53, Notch, Sonic hedgehog (Shh), bone morphogenetic protein (BMP), and Wnt (45, 58–64).

In murine airways, bronchioles terminate directly into alveolar duct openings at the bronchioalveolar duct junction and are populated by bronchioalveolar stem cells (BASCs) that exhibit transcriptional profiles of both airway secretory and ATII cells (SCGB1A1 and SFTPC) (41). Following distal lung injury, BASCs can differentiate into airway or alveolar epithelial cells (39) or to proximal epithelial cells after airway-specific injury (40–42). Separately, rare ITGB4β^+^H2-K1^hi^ progenitor cells located in proximal airways were shown to engraft into bleomycin-injured mouse lungs following intratracheal transplantation with subsequent differentiation into ATII cells (43, 44). Intralobular serous cells that coexpress SCGB3A2^+^SCGB1A1^+^ and KRT5, a marker of airway basal stem cells, were identified in an influenza acute lung injury model and may contribute to bronchiolization in IPF (45–47). These observations suggest that the plasticity of existing progenitor cells localized at the site of injury and the migration of anatomically distant epithelial cells following injury may dictate normal versus excessively fibrotic repair outcomes.

In humans, terminal respiratory bronchioles and alveolar ducts are separated by structures called respiratory bronchioles that contain airway, alveolar, and BASC-like cells. Among these, airway epithelial progenitors termed terminal airway secretory cells (TASCs, marked by SCGB3A2^+^SFTPB^+^) (48), respiratory airway secretory cells (RASCs, marked by SCGB3A1^+^SCGB3A2^+^SFTPB^+^CEACAM6^+^) (49, 50), and AT0 cells (marked by SCGB3A2^+^SFTPB^+^SFTPC^+^) (50) were identified as cell types of interest (Figure 2). Loss of anatomical structures in humans, such as terminal respiratory bronchioles and bronchoalveolar ducts that house the newly identified TASC/RASC populations, may play a significant role in the aberrant repair process that occurs in fibrosis (16). Recent work has demonstrated a loss of progenitor ATII cells and an increase in the number of BASCs during aging (65). It will be critical for the field to address the initial role and eventual loss of these progenitor populations in IPF. The role of genetic risk variants and/or aging in the generation of a vulnerable epithelium and potential consequences for the differentiation trajectory of these cells in vivo are incompletely understood. However, in patients with IPF, and especially those with the *MUC5B* promoter variant, MUC5B is ectopically expressed in the respiratory bronchiole (11, 66), a region of the lung that does not normally express MUC5B (67). This suggests that MUC5B may influence the cellular composition of these localized fibrotic regions of the lung. Furthermore, in vivo and human studies that examine the role of these newly identified progenitor populations during fibrosis development and in the context of a vulnerable lung epithelia are still needed, as much of our current knowledge stems from in vitro differentiation experiments. These should be complemented with studies in higher-order animals, including ferrets and pigs, as these species contain respiratory bronchioles with similar cellular composition and morphology found in the human lung (68).

### MUC5B and host defense.

In animal models and humans, MUC5B is essential for respiratory tract host defense (69–71). This requirement is met by tissue- and cell type–specific restriction of MUC5B to the tracheobronchial airways and submucosal glands, where cells are programmed to handle its biosynthesis and secretion. In bronchioles, MUC5B is produced by surface epithelial club cells, albeit at much lower levels than in bronchial epithelia. In unaffected individuals, MUC5B is undetectable in the most distal terminal and respiratory bronchiolar airways (67, 72–74). Presumably, restricted expression of MUC5B normally limits its accumulation in the distal airspace where it could interfere with particle clearance or gas exchange (29). Ectopic expression of MUC5B in terminal and respiratory bronchiolar airways in patients with IPF, especially in those with the *MUC5B* promoter variant (11, 66, 75), is thought to disrupt lung homeostasis and promote fibrotic remodeling in these vulnerable distal regions of the lung.

The *MUC5B* gene is 39 kb in length and encodes a 5,762–amino acid protein (596 kDa) that presents intrinsic challenges to cellular proteostasis. Mucins are secretory proteins that are targeted to the ER for translation, folding, and stabilization via disulfide bond formation. MUC5B contains more than 100 disulfide internal bonds per molecule at its amino (N-) and carboxy (C-) termini (76, 77). Furthermore, its N- and C- termini are separated by an approximately 3,000–amino acid stretch of unstructured domains fated to be O-glycosylated in the Golgi. Accordingly, MUC5B synthesis evokes high levels of steady-state ER stress, and mucous cells have adapted processes to dampen activation of an unfolded protein response (UPR). Polymeric mucin production has been best studied in the context of the IRE-1 pathway. Unfolded proteins stimulate IRE-1 ribonuclease activity to remove a normally unspliced intron in XBP1, enabling translation of a transcription factor that upregulates corrective and cytotoxic ER stress responses (78, 79). Importantly, IRE-1 has both a ubiquitous isoform (IRE-1α) and a mucous cell–specific isoform (IRE-1β) (78–83). The β isoform exhibits higher thresholds for activation, lower levels of XBP1 activation, and suppresses IRE-1α–mediated UPR activation to help maintain a sustainable ER stress response during homeostasis. Importantly, mucous cells exploit this through transcription factors such as SAM pointed domain-containing ETS transcription factor (SPDEF), which coordinately regulates expression of IRE-1β (84), mucous cell chaperones (85), and mucins themselves (86–89). To minimize ER stress and restore proteostasis, cells initiate an UPR (78). The UPR provides graded responses to ER stress by decreasing ER protein levels, improving folding, degrading proteins that cannot be corrected, or ultimately shifting cells toward senescence and apoptosis (90, 91). MUC5B misexpression alone could elicit UPR signals (e.g., apoptosis) in cells lacking IRE-1β resulting in tissue damage, which has been shown to occur in distal IPF lung epithelia (11, 66, 75, 92, 93). Additional work is needed to validate the regulation of mucous cell proteostasis regulators as well as IRE-1α– versus IRE-1β–dependent UPR activation in cells ectopically expressing MUC5B in IPF.

### Cell adhesion.

Cell-cell and cell-matrix contacts are critical for tissue integrity and host defense (94). Dysfunction of cell-cell and cell-matrix adhesion molecules (including desmoplakin [DSP], E-cadherin, integrins, and focal adhesion kinase [FAK]) affecting epithelial cells and myofibroblasts plays a pivotal role in the pathogenesis of IPF (95). Genetic variants of DSP are associated with IPF (Table 1) (96, 97). DSP facilitates cell adhesion in bronchial and alveolar epithelial cells, with high expression in basal cells (98), and enables cell migration, proliferation, and differentiation (99). Its dysregulation may promote progression of lung fibrosis through multiple aspects of decreased cell adhesion and disrupted tissue integrity. E-cadherin, a key component of adherens junctions, helps maintain epithelial barrier integrity through homophilic interactions between adjacent epithelial cells. In pulmonary fibrosis, decreased E-cadherin expression compromises cell-cell interactions, leading to impaired barrier function and eventual epithelial cell detachment from the basement membrane (100). Integrins are transmembrane receptors linking the extracellular matrix (ECM) to the intracellular cytoskeleton, playing a dynamic and crucial role in cell adhesion and signaling. TGF-β, a known mediator of fibrotic processes, is secreted into the microenvironment in a latent inactive form bound to latency associated protein and is activated by the binding of integrin αvβ6 (101–103). Inhibition of integrin αvβ6 in murine models of pulmonary fibrosis, including radiation- and bleomycin-induced injury, was shown to prevent lung fibrosis (104–106). Finally, FAK, a downstream effector of integrin signaling, has also been shown to regulate cell adhesion, migration, and survival of epithelial cells and differentiation and migration of myofibroblasts (107–109). In mice, small-molecule inhibition of FAK prevented bleomycin-induced lung fibrosis (109), while ATII-specific deletion of FAK following bleomycin-induced fibrosis resulted in ECM alterations, fibroblast activation, and inhibition of ATII cell apoptosis, suggesting a complex signaling dynamic between epithelial cells and fibroblasts (107). Understanding the intricate interplay between adhesion molecules and signaling pathways and if these are altered in a vulnerable host will be essential for developing targeted therapies to restore normal cell-cell and cell-matrix adhesions in fibrosis.

### Alveolar homeostasis and injury.

ATII cells play a crucial role in maintaining alveolar homeostasis by producing surfactant and serving as progenitor cells that regenerate damaged alveolar epithelium. In injured and fibrotic lungs, ATII cell function is impaired, leading to disrupted surfactant production and ineffective regeneration. In addition, mutations in several genes (*SFTPC*, *SFTPA1*, *SFTPA2*, *ABCA3*, and *NKX2-1*) that are uniquely expressed, or highly enriched, in ATII cells have been identified in patients with IPF (110–112). These genes are critical for alveolar epithelial cell specification (*NKX2-1*), surfactant homeostasis and function (*ABCA3*, *SFTPC*, *SFTPA1*, and *SFTPA2*), and innate immune responses (*SFTPA1* and *SFTPA2*), all of which work in concert to decrease surface tension within alveoli and defend against respiratory pathogens. Impaired surfactant composition and function resultant from loss-of-function mutations in surfactant-associated genes leads to alveolar instability and atelectasis (12, 113). Alveolar collapse has been reported in the unaffected parenchyma of IPF diseased lungs and has also been associated with IPF progression (114, 115). Additionally, it was shown that overexpression of the profibrotic factor TGF-β1 suppresses expression of surfactant proteins in ATII cells, leading to alveolar collapse prior to fibrosis in the bleomycin mouse model (116). Thus, alveolar collapse can contribute to early pathogenesis of IPF and may worsen upon activation of mesenchymal signaling.

N-terminal truncation mutations in surfactant protein C (*SFTPC*) lead to retention of SFTPC in endolysosomal compartments and aggresome formation (117, 118) and have been reported to be associated with IPF (113, 119–121). Transgenic expression of an *SFTPC* exon 4 truncation mutant (termed delta exon 4) in mice led to an embryonic lethal phenotype associated with high levels of transgenic protein, ER stress, and disrupted lung development (122). Expression of a different *SFTPC* variant (L188Q) in transgenic mice was not sufficient for the development of spontaneous fibrosis but augmented bleomycin-induced fibrosis (123). In vitro studies demonstrated that, while both the delta exon 4 mutant and the L188Q mutant induced ER stress and IL-8 secretion in A549 cells, only the delta exon 4 mutant was sufficient to activate NF-κB signaling (124). This demonstrated that expression of misfolded SFTPC protein and subsequent ER stress responses was sufficient to drive increased inflammatory signaling in ATII cells (123). To correct for the embryonic lethality and hypomorphic complications of these mutants, conditional knockin SFTPC-transgenic mice have also been created (I73T and the BRICHOS mutant C121G), which demonstrated both ER stress and spontaneous lung fibrosis (12). Confirmation of alveolar epithelial ER stress as causative for spontaneous fibrosis was demonstrated through conditional deletion of the *HSPA5* gene (encoding GRP78, a molecular chaperone necessary for inhibition of ER stress signaling). Mice with ATII-specific deletion of GRP78 developed ER stress and spontaneous pulmonary fibrosis, establishing a link between ER stress and fibrotic lung disease (125). Moreover, inhibition of IRE-1α reduced ER stress and lung fibrosis in *Sftpc*^c121g^ mice (93).

### Telomere attrition and cell senescence.

While lung epithelial cells can have relatively long half-lives (126), epithelial cell senescence may be accelerated by a number of aging-related events, including DNA damage (127), telomere attrition (128), dysregulated proteostasis (125, 129–135), and mitochondrial stress (136). At a molecular level, senescence is a state of irreversible replicative arrest characterized by markers of DNA damage, cellular hypertrophy, upregulation of lysosomal β-galactosidase, and expression of the cyclin-dependent kinase inhibitors (*CDKN1A* and *CDKN2A*) (137). Lung epithelia in IPF express CDKN1A (138–140) and its paralog, CDKN2A (141, 142). Moreover, deletion of CDKN2A^+^ senescent cells was protective in murine bleomycin-induced fibrosis (32).

Additional evidence for senescent and aging-related phenotypes in IPF comes from known associations between rare mutations in genes that encode enzymes responsible for maintaining DNA integrity. Telomeres are segments of chromosomes that enable DNA repair machinery to discriminate between chromosomal ends and DNA double-strand breaks (143), and breakdown in telomere maintenance triggers cellular senescence (144–148). Telomere shortening is associated with IPF (149, 150), and it is a common finding in IPF lung epithelia (138) and peripheral blood mononuclear cells (151). Genetic variants in the telomere synthesis enzymes *TERT* and *TERC* (144, 152) have also been implicated in IPF (150, 153–158), and sporadic mutations in telomere-supporting shelterin proteins have also been found to be associated with IPF (159–161). Continued replication after telomere attrition requires telomere lengthening to prevent chromatin erosion (144, 162, 163). Haploinsufficiency of telomere maintenance complexes is sufficient to promote intergenerational telomere attrition and development of myelofibrosis and pulmonary fibrosis in dyskeratosis congenita (151, 164–166).

### Epigenetic regulatory mechanisms in IPF epithelia.

Emerging transcriptomic data have defined abnormal basaloid cells as a characteristic attribute of IPF epithelia (55), and the relatively stereotypical transcriptomic features of this pathogenic basaloid cell population implicate cellular memory as a likely contributing mechanism. Transcription-based cellular memory, which arises as a consequence of autoregulated transcription factors and positive feedback circuits, is a well-described driver of lineage commitment during normal development (167), and these factors are increasingly associated with various diseases (168, 169). Cellular memory mediated by metastable transcription circuits is thus a potential contributor to both normal basal cell programming and the misprogramming of basaloid cells in IPF. As an additional, and potentially reinforcing mechanism, widespread changes in DNA methylation in whole lung tissue (170) and fibroblasts (171) have been associated with IPF. DNA methylation (172, 173); histone modifications, including methylation or acetylation; and other forms of chromatin remodeling (172, 174, 175) may play a critical role in ectopic expression of MUC5B with or without the promoter variant risk allele and in further stabilizing the aberrant basaloid cell fate. Additional understanding of how epigenetic changes and transcriptional circuits affect progenitor epithelial populations and how this cellular memory contributes both to vulnerability and disease progression is needed.

## Bronchoalveolar epithelia, honeycomb cysts, and fibrosis

Bronchiolization of the distal airspaces and loss of small airways have been appreciated as features of the IPF lung for nearly five decades (176). Until recently, however, the mechanisms driving these cellular and structural changes and their effect on patient survival and disease progression remained unclear. Initial work determined that bronchiolization and honeycomb cysts were characterized by their remarkable similarity to the airway epithelium (11, 177) and that basal cell–related gene signatures from bronchoalveolar lavage of patients with IPF predicted significantly worse mortality (178). Recent work has begun to elucidate the cellular origins underlying bronchiolization and cyst formation, demonstrating the capacity for aberrant alveolar epithelial differentiation following injury to drive cyst formation (179, 180). Separately, it has been shown that primary human distal airway epithelial cells derived from samples from patients with IPF possess a biophysically distinct YAP-dependent collective migratory phenotype, distinguishing them from their healthy counterparts (181). Ex vivo live imaging of injured murine airways demonstrates a conservation of this YAP-dependent migratory program that is likely important in bronchiolization and cyst formation (181, 182). Additionally, YAP signaling has been shown by multiple groups as a critical regulator of ATII cell proliferation and ATI cell differentiation (182–186).

### Ectopic MUC5B expression drives distal lung pathologies.

A key, currently unanswered question is whether, and if so to what extent, ectopic expression of MUC5B in bronchiolar epithelia of patients with IPF contributes to persistent, progressive fibrosis and to the formation of honeycomb cysts. Transgenic mice expressing increased levels of *Muc5b* in the distal airways (ectopic expression under the *Scgb1a1* promoter) or alveoli (ectopic expression under the *Sftpc* promoter) fail to spontaneously develop fibrosis or honeycomb cysts (29). However, when transgenic *Scgb1a1-Muc5b* mice are injured repetitively with bleomycin, both fibrosis and microcyst formation are enhanced and prolonged (29, 187). These findings suggest that fibrosis and honeycomb cysts develop in a vulnerable lung (potentially driven by MUC5B ectopic expression) after a repetitive secondary hit that reprograms a vulnerable epithelium (Figure 2). Current findings suggest that the profibrotic effect of MUC5B ectopic expression in distal airway cells on fibroblasts may be indirect, possibly mediated by exacerbation of epithelial injury and destruction that provides an altered “substrate” or “niche” onto which lung fibroblasts migrate, gain resistance to apoptosis, persist, and continue to express and deposit fibrotic ECM (182). Thus, it will be critical to understand how excess MUC5B influences molecular drivers that can elicit a profibrotic phenotype from the underlying mesenchyme. This includes YAP signaling, which has been shown by multiple groups as a critical regulator of ATII cell proliferation and ATI cell differentiation (182–186) and well-known signaling cascades (e.g., EGFR/YAP/SRC) and novel pathways (e.g., IL-6 and IL-11) (101, 181, 182) implicated in disease initiation and progression.

### Fibroblast heterogeneity during homeostasis and injury.

The alveolar walls and septa of healthy lungs contain resident PDGFRα^+^ alveolar fibroblasts that synthesize components of the ECM (188) and serve as niche cells that support the growth and function of ATII cells by secreting instructive factors required for ATII cell survival and proliferation (e.g., IL-6, FGF-7, Wnt) (189) and transfer phospholipid precursors from alveolar capillary endothelial cells to ATII cells (190, 191). A smaller number of PDGFRβ^+^ pericytes are located in alveolar walls and provide trophic support to alveolar aerocytes and general capillary endothelial cells (190, 191). In addition, PDGFRβ^+^ pericytes and adventitial fibroblasts surround distal airways and blood vessels. In the normal adult lung parenchyma, contractile α smooth muscle actin–expressing (α-SMA–expressing) myofibroblasts are found to extend from conducting airways out to alveolar ducts and are known as ductal myofibroblasts (192). These spatially distinct fibroblast subsets exhibit overlapping and distinct gene expression patterns that collectively contribute to their function in healthy lungs.

In response to injury and loss of ATI and ATII cells (36, 193), lung fibroblasts are rapidly mobilized and actively contribute to lung repair and regeneration. scRNA-sequencing studies in bleomycin-instilled PDGFRα-GFP and COL1A1-GFP reporter and lineage-traced mice have shown that PDGFRα^+^ fibroblasts and PDGFRβ^+^ pericytes/adventitial fibroblasts migrate, proliferate, and accumulate in bleomycin- and influenza virus–injured lungs (194–196) and become reprogrammed to express profibrotic ECM (e.g., COL1a1, SPP1, FN1, ELN) and contractile proteins (e.g., α-SMA, CNN1, TAGLN) (101, 180, 196, 197). These studies also identified novel profibrotic genes and transcription factors that differentiate newly identified profibrotic fibroblast subpopulations, including *CTHRC1*, *THRC1*, *RUNX1,* and *SFPR1* (195, 196). A specific lung fibroblast, the alveolar fibroblast, appears to be critical to alveolar homeostasis and when stimulated with either IL-1α or TGF-β can develop into inflammatory or fibrotic fibroblasts (198). As repair continues, some of these profibrotic fibroblasts undergo apoptosis and are cleared, while those remaining (and potentially newly proliferated or migrated fibroblasts) undergo further reprogramming to express genes involved in lung development and repair (195). Together, the enrichment of these later pathways support ATII proliferation and differentiation into ATI cells, while complementary angiogenic pathways contribute to the regeneration of alveolar capillary endothelium. Finally, excess ECM is degraded, leading to restoration of lung architecture and function. We have referred to this resolution phase, which is initiated by the wave of fibroblast apoptosis, as “homeostatic fibrosis resolution” (195).

Reciprocal interactions between fibroblasts and epithelial cells within the alveolar niche are critical for homeostasis of the lung parenchyma. Seminal coculture studies have demonstrated that primary rat, mouse, and human ATII cells from nondiseased donor lungs suppress fibroblast proliferation via an autocrine signaling loop, wherein IL-1α derived from ATII cells activates COX2-dependent prostaglandin E2 (PGE2) secretion from fibroblasts that inhibits their proliferation (199–202). PGE2 synthesis is reduced in bronchoalveolar lavage fluid from patients with IPF (203), suggesting that perturbation of this ATII fibroblast signaling loop, after ATII cell injury and/or apoptosis, contributes to exuberant fibroblast proliferation in the fibrotic lung. In addition, injured ATII cells from IPF lungs show increased expression of CTGF (200), TGF-β (204–206), and Shh (207–209), all of which stimulate fibroblast proliferation and induce collagen secretion and α-SMA expression. Although critical crosstalk between fibroblast subpopulations and distal basal cells has been demonstrated in organoid cultures (50), further studies with the newly identified progenitor epithelial cell populations in the distal airways (AT0, TASC, and RASC) in vivo remain to be conducted.

### Fibroblast heterogeneity during fibrosis.

A central pathologic feature of IPF and in fibrotic mice is the persistence of nonproliferating, apoptosis-resistant, and often senescent α-SMA^+^ and ECM-producing profibrotic fibroblasts (Figure 2) (210–214). These arise though multiple mechanisms, including increased resistance to apoptotic signals, and lead to a persistently activated profibrotic fibroblast population that promotes disease progression through unabated aberrant ECM production and eventual senescence (215) (Figure 2). Recent studies in mouse fibrosis models and human IPF tissue demonstrate an interaction between the development and persistence of senescent KRT8^+^ transitional basaloid epithelial cells and profibrotic fibroblasts (56, 61, 216). Often occurring at the edge of the fibroblastic foci, these cell populations are thought to represent active areas of fibrotic destruction in the lung (217). Fibroblastic foci contain discrete areas of fibroblasts, myofibroblasts, and newly formed collagen in humans. They have been shown to dissociate capillary vessels from the alveolar epithelium, disrupt normal basement membranes, and induce a transitional epithelial cell phenotype that lines the foci and results in the loss of normal alveolar septa (218). ECM in the fibrotic lung is stiffer and results in generation of greater mechanotransductive forces in fibroblasts and remodeled and aberrant epithelial cells. This is driven by the well-described positive feedback amplification loop involving expression and activation of TGF-β. This self-perpetuating circuit, which is reminiscent of the interactions described above between fibroblasts and ATII cells in healthy lungs, supports fibrotic fibroblasts and transitional basaloid cells and promotes continual disruption of epithelial barrier integrity. It also inhibits appropriate epithelial cell differentiation and aberrant fibroblast ECM production and organization, contributing to persistent and progressive disease (56, 219).

## Therapeutic implications

To date, there are two approved therapies for IPF, nintedanib and pirfenidone (220, 221), which slow the progressive loss of lung function but are not curative and have side effects that limit their efficacy (222). Furthermore, patients continue to decline despite these medications and subsequently develop end-stage lung disease (223). Thus, it becomes imperative to look beyond typical antifibrotic signaling pathways for innovative therapeutic directions.

### Novel targets in the clinic and on the horizon.

Genetic and nongenetic risk factors have identified several mechanisms that appear to be critical to the development of IPF and may help to identify patients at earlier disease stages. Currently, these mechanisms focus on the respiratory bronchioles and alveolar epithelia and include dysregulation of host defense, cell adhesion, telomere attrition, stem cell exhaustion, early cell senescence, and dysfunctional surfactant protein biology. This suggests that genetic variants associated with unique genes converge on specific pathways that disrupt terminal respiratory and alveolar epithelial structure and function. For example, in those with the gain-of-function *MUC5B* promoter variant, approaches that decrease MUC5B expression could reduce the vulnerability of the lung by improving mucociliary clearance and/or ameliorating chronic ER stress. In individuals with telomerase mutations, the accelerated rate of cell senescence could be slowed by targeting DNA repair, reducing oxidative stress, or by using senolytic agents to selectively induce death of senescent cells. A randomized phase IIa clinical trial for IPF with an anti-αvβ6 integrin monoclonal antibody has recently demonstrated a reduction of TGF-β signaling. The phase IIa INTEGRIS-IPF trial using an αvβ6 integrin small-molecule inhibitor slowed the rate of forced vital capacity decline (224, 225). Recent preclinical studies in mice have shown that therapeutic targeting of BCL-2 and its related family members, BCL-XL and BCL-W, with the BH3 mimetic drug ABT-263 (Navitoclax) reduced the severity of silica- and bleomycin-induced pulmonary fibrosis (213, 226–228) as well as scleroderma-like skin fibrosis in mice (229). Thus, the development of fibroblast resistance to apoptosis may prove relevant to persistent and progressive lung fibrosis. However, these antiapoptotic pathways may also be exploited for targeted elimination of profibrotic fibroblasts and reducing fibrosis in general. The concept of targeting therapy to specific gene variants and disease mechanisms has been successful in fields such as rheumatology and oncology and would represent an advance in the treatment of IPF.

### Gene editing.

Gene editing and gene therapy technologies have developed rapidly during the last several decades (230–232). Potential treatment targets in IPF include the *MUC5B* promoter or telomere gene variants, which are appealing due to the elevated expression of MUC5B (173) and shortened telomeres (233–236) in all individuals with IPF, regardless of a genetic mutation. Gene transfer has been the most frequent approach used to attenuate lung fibrosis in rodent models. AAV-based delivery of telomerase (*TERT*) to ATII cells of bleomycin-treated *TERT*-deficient mice increased telomere length, proliferation, and reduced inflammation and fibrosis (236). Downregulation of the proinflammatory cytokine milieu, affecting IL-6, IL-10, IL-17A, and IL-33, also reduced lung fibrosis (237–239). Other genes, many related to vascular homeostasis, delivered to injured lungs also reduced inflammation, fibrosis, and apoptosis (240–245). In humans, both viral and nonviral delivery methods have been advanced for lung targeting (232, 246, 247). However, all current approaches are limited by the complex human lung structure with multiple barriers to delivery, such as mucous, macrophage-mediated phagocytosis, and epithelial barrier function (248, 249). Cell-based approaches with genetic reprogramming and reengraftment may be an alternative approach for IPF gene therapy (250, 251); however, these approaches may be limited by low engraftment rates, likely due to lack of appropriate niche space in lungs with established fibrosis and off-target effects of systemic gene therapy (252–254).

### Epigenetic approaches to IPF therapies.

Epigenetic reprogramming of cells can provide reliable, long-lasting therapeutic effects in vivo (255). First-generation FDA-approved epigenetic therapeutics, such as azacitidine DNA methyltransferase inhibitors (i.e., Decitabine, Vidaza) and histone modifying drugs, have proven effective in treating diseases such as lung cancer (256, 257) but are broadly acting with profound side effects. More recently developed locus-specific epigenetic approaches to genome editing technologies hold promise in development of more effective and long-lasting epigenetic therapeutics (231). Several studies demonstrate that DNA methylation of the *MUC5B* promoter variant is associated with MUC5B expression (172, 173). Histone modifications, such as acetylation and chromatin remodeling, can also regulate *MUC5B* expression (172, 174, 175). Thus, modifications of epigenetic marks may prove beneficial in regulating *MUC5B* expression or other IPF risk genes, especially those associated with a gain or loss of function (258–262). Specific approaches to targeting the epigenome (255, 263–268) rely on modifying proteins to bind specific sequences in the genome by using CRISPR-deactivated Cas9 (dCas9) and related technologies. However, the main barriers for epigenetic reprogramming to become a therapeutic target for IPF remain efficiency of in vivo construct delivery into cell types of interest and the risk of side effects and nonspecific activity (252, 269, 270). Antibody drug conjugates, in which bioactive payloads can be delivered to specific cell types, have shown promise in oncology for reducing off-target effects (271). Epigenetic and other targeted cellular reprogramming efforts in IPF may be facilitated by this rapidly improving technology.

## The path ahead

The complex etiology and biology of IPF creates challenges and opportunities for the path forward. Delineating pathways that lead to host vulnerability, injury, or repair will identify those responses that initiate disease versus those that result in persistent and progressive lung fibrosis.”. While defining the temporal relationship pathologically among vulnerability, early disease, and persistent and progressive lung fibrosis is critical, these pathological stages of lung fibrosis will best be understood within the context of etiologic drivers. These dimensions of IPF, etiology and stage, should highlight the key pathologic pathways and address many of the unmet needs in this complex disease, including identifying sites of lung vulnerability, defining mechanisms that adversely disrupt epithelial function and activate fibroblasts and lead to lung remodeling, and characterization of high-priority targets for intervention. For example, genetic and nongenetic drivers of IPF have identified bronchiolar and alveolar epithelia as initial targets of injury.

Mechanisms through which epithelial progenitor cell populations, especially in the distal airspace, are injured or are unable to mediate repair need further investigation. New cell types/states are continuously emerging through single-cell and spatial transcriptomics that have distinct but often overlapping identities and functions. Whether similar antiproliferative and fibroblast activation signaling pathways are operative in newly identified progenitor epithelial cell populations in the distal airways (AT0, TASC, and RASC) of human lung remains to be determined. Thus, the identity, ontogeny, transcriptional programming, and temporal-spatial relationship of epithelial progenitors to lung fibrosis represent an area of investigation with clear relevance to injury, repair, and fibroproliferation.

Understanding the consequences of genetic risk variants for cell differentiation patterns of progenitors in vivo is imperative. A key, currently unanswered question is whether, and, if so, to what extent, ectopic expression of MUC5B in bronchiolar epithelia of patients with IPF contributes to persistent, progressive fibrosis and to the formation of honeycomb cysts. Understanding the intricate interplay between adhesion molecules and signaling pathways will be essential for developing targeted therapies to restore cell-cell and cell-substrate adhesion and halt the progression of pulmonary fibrosis and prevent lung remodeling.

Finally, applying this new knowledge to early recognition of disease before the onset of irreversible and progressive lung fibrosis and developing novel therapeutics directed at etiologic and temporal drivers of lung fibrosis will ultimately transform the care of patients with IPF from palliative to curative.

## Figures and Tables

**Figure 1:**
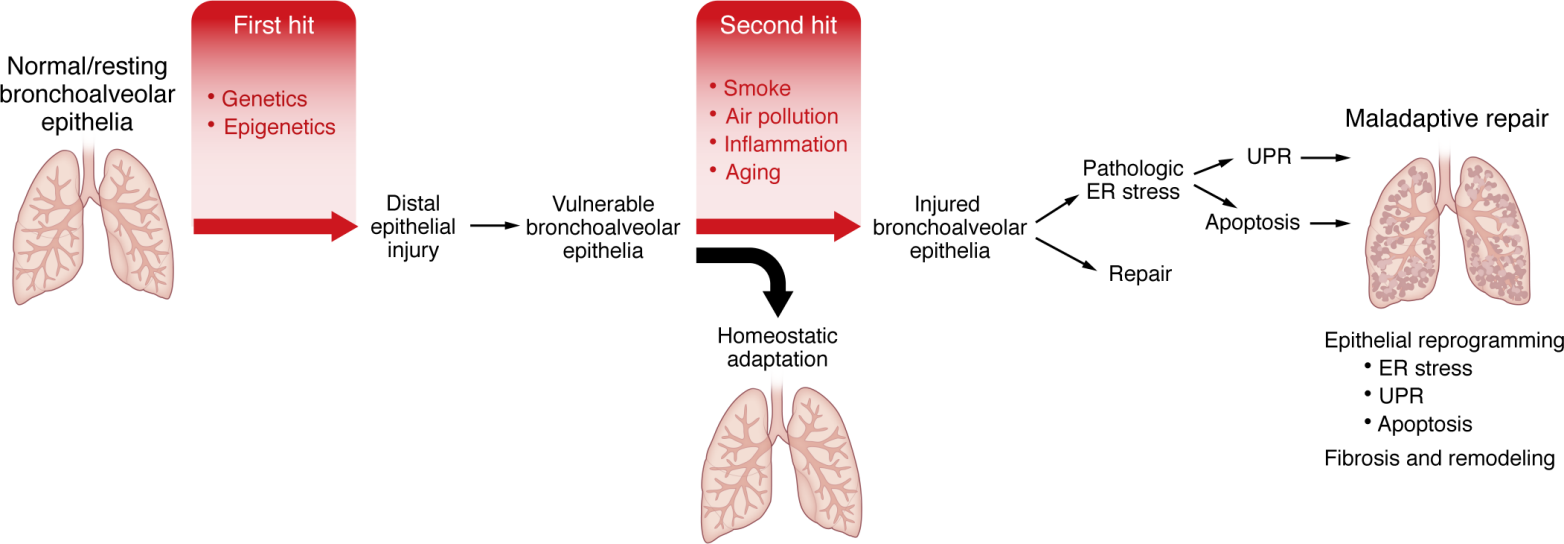
Two-hit model of pulmonary fibrosis. We postulate that genetic and epigenetic etiologic drivers establish a vulnerable bronchiolar and alveolar epithelia (first hit) and that this results in homeostatic adaptation without the development of lung fibrosis. Persistent and progressive lung fibrosis can be triggered by a second hit (such as tobacco smoke, air pollution, inflammation, and/or aging) to the bronchiolar and alveolar epithelia, resulting in epithelial reprogramming, endoplasmic reticulum (ER) stress, unfolded protein response (UPR), apoptosis, and ultimately leading to fibroblast accumulation and activation, fibrosis, and abnormal lung remodeling.

**Table 1:**
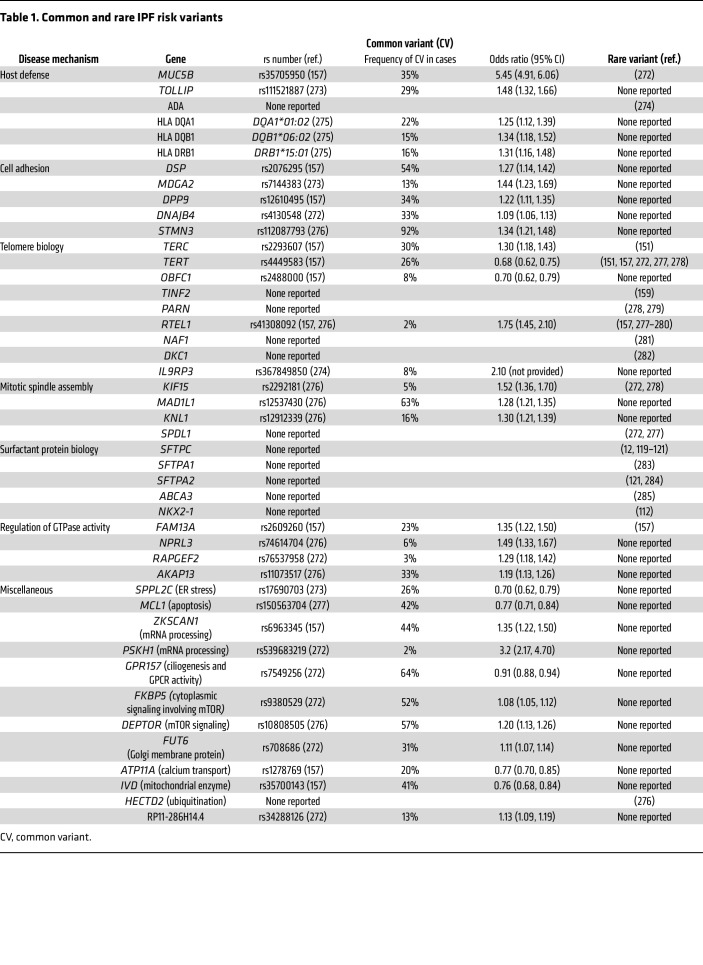
Common and rare IPF risk variants

## Version 1 01/02/2025

Electronic publication
